# Nanomaterials engineered for photothermal therapy in neural tumors and neurodegenerative diseases: biomaterial design, clinical mechanisms and applications

**DOI:** 10.3389/fbioe.2025.1631627

**Published:** 2025-07-21

**Authors:** Hanjing Zhu, Wei Yang, Yijun Suo, Ye Liu, Xinyi Zhan, Jun Zhou, Zhiying Chen, Xiangbing Wu, Xiaoping Yin, Bing Bao

**Affiliations:** Department of Neurology, Affiliated Hospital of Jiujiang University, Jiujiang, China

**Keywords:** photothermal therapy (PTT), glioma, neurodegenerative diseases, combination therapy, blood-brain barrier, heat shock proteins (HSP), organic nanoplatform, inorganic nanoplatform

## Abstract

The rising incidence of neural tumors and neurodegenerative diseases cause significant health, emotional, and financial burdens. Conventional treatments like surgery and chemotherapy often lack effectiveness. However, advancements in nanotechnology, particularly photothermal therapy (PTT), offer new hope. PTT is widely studied for neural tumors and neurodegenerative diseases due to its simplicity, rapid recovery, combined therapeutic potential, and compatibility with imaging techniques. This innovative approach could revolutionize the diagnosis and treatment of neural tumors and neurodegenerative diseases, addressing current limitations and improving outcomes. In this article, we offer a comprehensive overview of the rational design and engineering of various nanomaterials designed specifically for PTT applications in neural tumors and neurodegenerative diseases, including organic platforms such as liposomes, dopamine, etc. and inorganic platforms such as gold nanomaterials, carbon nanomaterials, etc. A comparative analysis of these platforms examines their biocompatibility and potential for biodegradation. It also assesses their manufacturing scalability, cost-effectiveness, regulatory challenges, and ultimate potential for clinical translation. We also update the therapeutic advances of PTT in neural tumors (Glioma, Peripheral nerve sheath tumors, Spinal metastases from *in situ* tumors and brain metastases) and neurodegenerative diseases (Alzheimer’s disease, Parkinson’s disease, Huntington’s disease), and systematically summarize the mechanisms of PTT application in neural tumors and neurodegenerative diseases. In the end, we provide an in-depth discussion of the advantages and disadvantages of PTT and the perspectives for its application in the above neurological disorders.

## 1 Introduction

PTT is a novel non-invasive treatment method, whose basic mechanism lies in the fact that with the help of specific photothermal transduction agents (PTAs), it can effectively convert the received photon energy into thermal energy under the irradiation of exogenous light such as near-infrared (NIR) ray, and the effect of this process is mainly reflected in the photothermal ablation of local tissues to exert its therapeutic effect. Currently, there is a high level of interest in research on the application of PTT in oncology (Cheng et al., 2014; Li et al., 2020; Zhao et al., 2021).

Photophysically, a PTA absorbs photon energy upon exposure to specific NIR light. Photon absorption elevates the PTA from its ground singlet state to an excited singlet state. Subsequently, the electronic excitation energy dissipates through vibrational relaxation. The excited PTA then reverts to its ground state via collisions with neighboring molecules. These molecular interactions generate heightened kinetic energy, inducing localized warming (Li et al., 2020). In essence, PTAs undergo lattice vibrations or electronic oscillations after absorbing electromagnetic radiation. Such activity increases the material’s temperature, creating a photothermal effect on the local environment (Wang Y. et al., 2021; Yu et al., 2024).

Different PTAs utilize distinct photothermal conversion mechanisms. Noble metal nanoparticles, like gold and silver, exhibit a unique localized surface plasmon resonance (LSPR) effect. Through LSPR, decaying surface plasmon oscillations radiate energy, converting absorbed light into heat. Semiconductors achieve photothermal conversion via two main pathways. One mechanism is free carrier absorption, analogous to the metallic LSPR effect. The second pathway involves the intrinsic absorption band gap between the material’s valence and conduction bands. For most carbon-based materials, their π bonds are generally weaker than σ bonds. This bond characteristic facilitates electron transitions under illumination, triggering polarization relaxation. Light energy subsequently transfers to crystal lattice vibrations, yielding photothermal conversion (Wang Y. et al., 2021; Yu et al., 2024).

Due to the limitations of PTT monotherapy in treating diseases, PTT is often combined with other nanoplatform-based therapies, such as Chemotherapy (CT), radiotherapy (RT), photodynamic therapy (PDT), Chemodynamic Therapy (CDT), Sonodynamic Therapy (SDT), gene therapy, immunotherapy, etc. to achieve greater synergistic effects (Liu et al., 2019b; Xie et al., 2020; Qiao et al., 2022). PTT is increasingly favored by researchers for its simplicity, short treatment time, rapid recovery, and excellent light-controlled drug release on combination chemotherapy (Cheng et al., 2014; Zhao et al., 2021).

With the growing interest in nanomedicine and its wide application in neurological diseases, this review focuses on the progress of PTT with nanomaterials, which has been rapidly developed in recent years for application in neural tumors and neurodegenerative diseases. We aim to provide a comprehensive overview of current strategies for rational design and engineering modification for multiple nanomaterial platforms, covering both organic (e.g., liposomes, dopamine-based nanomaterials) and inorganic (e.g., gold nanomaterials, carbon-based nanomaterials) materials for specific applications in neurological diseases. In particular, the article compares the organic and inorganic platforms in terms of their biocompatibility and biodegradation, manufacturing scalability and cost-effectiveness, and regulatory challenges and potential for clinical translation. We emphasize how to develop targeted material designs based on the unique pathophysiological features and biological barriers of the nervous system. Subsequently, the article updates its latest research progress in specific diseases, including glioma, peripheral nerve sheath tumor, metastatic neurological tumors, as well as typical neurodegenerative diseases such as Alzheimer’s disease (AD), Parkinson’s disease (PD), Huntington’s disease. We also systematically summarizes the clinical mechanisms by which PTT exerts its efficacy in the treatment of neurological disorders, involving a range of signaling molecules and pathways for apoptosis, etc. By comprehensively analyzing the material strategies in these different diseases, and comparing the strengths, weaknesses and adaptations of various types of nanoplatforms, we seek to provide a broad and in-depth perspective to reveal how material innovations can drive the translational potential of PPT in the treatment of major neurological diseases.

## 2 Nanomaterials engineered for PTT of neural tumors and neurodegenerative diseases

PTAs commonly used in PTT are broadly classified into organic and inorganic nanomaterials. Among inorganic nanomaterials, gold nanomaterials, carbon nanomaterials, palladium nanosheets, copper sulfide nanoparticles, and some other newly reported materials, such as black phosphorus-based materials, are commonly used; organic nanoparticles include protein-containing near-infrared absorbing conjugated polymers, porphyrins, liposomes, and nanomicelles encapsulated with near-infrared dyes (Cheng et al., 2014; Huang et al., 2021; Overchuk et al., 2023). In the following section, nanomaterials currently used in PTT applied to neural tumors and neurodegenerative diseases will be discussed mainly according to the classification of organic and inorganic materials.

### 2.1 Neural tumors

#### 2.1.1 Organic nanoplatform

##### 2.1.1.1 Liposome nanoparticles

Liposomes are nanoscale vesicles primarily composed of lipid or phospholipid molecules. Owing to their nanosize, biocompatibility, and biodegradability, liposomes have demonstrated potential in nanomedicine alongside broader applications in cosmetics and food industries (Panahi et al., 2017).

The liposomes’ stability and effective photothermal conversion capabilities also enable simultaneous fluorescence and photoacoustic imaging (PAI) (Zeng et al., 2023). Microfluidics (e.g., lab-on-a-chip platforms) facilitates the precise preparation of liposomes, which was used by Cao et al. to prepare vitexin/indocyanine green (ICG) liposomes, thereby successfully enhancing the poor solubility of vitexin in water, while improving the uniformity and dispersion of the particles. The resulting liposomes promoted combined photothermal and photodynamic effects (Cao X. et al., 2023). Liposomes can also be designed as responsive nanoprobes. Chen et al. designed a Lipo@HRP&ABTS nanoprobe. In which Horseradish peroxidase (HRP) catalyzed the oxidation of ABTS by H2O2 to produce a green derivative with strong near-infrared absorbance. With systemic administration, the nanoprobe detects hydrogen peroxide produced by tumors, enabling *in vivo* PAI to visualize small (∼2 mm) subcutaneous tumors and gliomas *in situ* (Chen et al., 2017).

##### 2.1.1.2 Polydopamine nanoparticles

Polydopamine (PDA) is an oxidative self-polymerized form of dopamine under alkaline conditions, originally inspired by the adhesive properties of mussel foot proteins containing catechol and lysine. Developed in nanoparticulate form in 2009, PDA exhibits melanin-like properties, excellent biocompatibility, aqueous solubility, and strong near-infrared absorption for PTT applications (Poinard et al., 2018).

Dube et al. prepared fluorescent poly-levodopamine nanoparticles (FLs) incorporating levodopa, DHI, and DHICA units. Self-assembly yielded diverse species possessing energy gaps from UV-visible to NIR, leading to broad absorption and excitation-dependent emission. The incorporated levodopa unit aids nanoparticle penetration across the blood-brain barrier (BBB). Leveraging the fluorescence and BBB-penetrating capabilities, multifunctional nanoplatforms co-loaded with doxorubicin (DOX)/ICG/peptide, termed FLDIPs, were developed (Dube et al., 2021). PDA also enables synergistic therapeutic approaches. For instance, nanoparticles radiolabeled with ^211^At and functionalized with a fibroblast activating protein inhibitor (FAPI) impede U87MG cell proliferation via combined photothermal and targeted alpha therapy. Observed cytotoxicity vanished upon omission of either ^211^At labeling or PTT activation (Li F. et al., 2024). Furthermore, surface engineering has optimized PDA vectors for glioblastoma (GBM). PDA nanoparticles underwent modification with the IL-13Ra2 ligand Pep-1. Resulting particles exhibited strong affinity for U87 cells and enhanced penetration into them. Peptide functionalization was demonstrated to facilitate BBB and cell membrane traversal, enabling efficient nuclear delivery; conversely, unmodified nanoparticles were confined to the cytoplasm (Wu et al., 2023).

##### 2.1.1.3 Peptide nanoparticles

Peptide-based nanomaterials, encompassing cyclic peptides, aromatic dipeptides, amphiphilic peptides, and polypeptides, are engineered into supramolecular nanostructures with adjustable characteristics. Possessing inherent biocompatibility, peptide nanomaterials can also modify photosensitizer behavior via molecular interactions. Their ability to modulate photosensitizer activity allows for precise control over photothermal effects. Consequently, such systems hold significant potential for cancer PTT (Abbas et al., 2017).

Engineered peptide nanoplatforms exhibit enhanced functionality beyond simple drug delivery. There are nanoparticles that enable precision diagnostics, providing features such as dual-modal contrast imaging and efficient photothermal ablation, and incorporate a self-sensing mechanism based on Förster resonance energy transfer (FRET), this allows the therapeutic process to be visualized *in situ* via corresponding signaling changes, thereby guiding precise PTT (Hu et al., 2018). Other designs promote active tumor targeting and fluorescence imaging (FLI), which facilitates tumor margin detection to guide precision surgery and local PTT (Jia et al., 2019).

Strategies have also improved therapeutic impact and delivery. Dube et al. developed multicomponent ICG-loaded peptide co-assembled nanoparticles. These engineered nanoplatforms exhibited significantly higher collective CT-PDT-PTT efficacy when compared to free, discrete therapeutic fractions (Dube and Panda, 2023). Self-assembled systems, using a phenylalanine peptide core responsive to tumor glutathione conditions, enhanced retention within the tumor. Such constructs achieved significant GBM inhibition in the U87MG-luc model, with an endpoint tumor volume 2.61-fold smaller than that of controls (P < 0.01) (Song W. et al., 2023). Selectivity has been addressed through surface modifications. Zhuge et al. produced indocyanine green-loaded silk fibroin nanoparticles (ICG-SFNPs) and their chitosan-modified variants (ICG-CSF). *In vitro* experiments, a small number of ICG-CSFNPs were phagocytosed by RAW264.7 macrophages, whereas C6 glioma cells were susceptible to internalization, leading to significant toxicity to these tumor cells after NIR irradiation (ZhuGe et al., 2019).

Specific organic nanomaterials, including dyes and polymers, are applied in PTT for neural tumors. For instance, lipoprotein-mimicking nanoparticles can improve tumor selectivity. Modifying liposomes with ApoE peptides enhances drug delivery and therapeutic efficacy while reducing side effects (Huang et al., 2024).

Novel delivery mechanisms are also under investigation. Doxorubicin-loaded apoptotic bodies are phagocytosed by monocytes/macrophages. Guided by tumor chemokines, these cells infiltrate the glioma, delivering their payload via a “hitchhiking” effect (Liu Y. et al., 2023). Similarly, engineered exosomes provide a biomimetic platform. One design features a microglia-derived exosome shell for targeting and a core with AIE agents. This structure enables mild photothermal therapy (MPTT) and NIR-II fluorescence for glioblastoma therapy (Lin et al., 2023).

Polymer-based nanostructures also show promise. PA1094T nanoparticles combine mild PTT with pyroptosis to augment anti-tumor immunity (Li et al., 2025). Other polymers containing tetraphenylene (TPE) leverage its unique molecular structure. Intramolecular rotation within TPE facilitates heat release, while its twisted conformation promotes strong intramolecular charge transfer in the conjugated copolymer. The interplay of these TPE characteristics enhances photothermal capabilities (Su et al., 2025).

#### 2.1.2 Inorganic nanoplatform

##### 2.1.2.1 Gold nanoparticles

Gold nanoparticles (AuNPs) attract significant research interest owing to their LSPR. LSPR originates from the collective oscillation of conduction electrons when exposed to NIR light, enabling effective conversion of photonic energy into heat. The optical behavior of AuNPs is governed by their morphology and the surrounding dielectric medium. Variations in morphology and medium could influence LSPR characteristics, such as absorption and scattering efficiency, and cause spectral peak shifts under changing surface conditions (Turkmen Koc et al., 2024).

Duan et al. researchers found that gold nanorods (Au NR) in CD-PGEA structures have inherent photoacoustic and X-ray computed tomography imaging capabilities for deep tissue detection, in addition to mediating PTT. The addition of fluorescent quantum dots (QDs) further enhances the sensitivity of both imaging modalities (Duan et al., 2017). The targeting strategy involves combining biotin with gold nanoparticles. This helps the nanoparticles move to the cellular site and promotes their effective binding to tumor cells (He et al., 2021).

Specific morphologies offer distinct benefits. Hollow gold nanoparticles (HGNP), synthesized at higher gold concentrations, exhibit low porosity and absorb at lower wavelengths. HGNP demonstrate resilience against repeated NIR irradiation. Combining HGNP/NIR with DDTC-Cu for PTT achieved significant *in vitro* cytotoxicity (20%). Such synergy surpassed the outcomes of NIR or the DDTC-Cu/HGNP mixture alone (Liu et al., 2024b). Hu et al. described ARCR, derived from 2D nanomaterials. Possessing large surface areas, ARCR strengthen surface plasmon resonance within the 650–1,350 nm biological window. Tips and small branches on their dendritic structures facilitate rapid heat transfer. Consequently, both PTT and PAI efficiency are significantly improved. Separately, siRNA-loaded AuNSs markedly inhibited polo-like kinase 1 oncogene expression (Hu et al., 2024). MA et al. developed AMMD featuring a complex double-layer porous architecture. Its internal metal-organic framework (MOF) component encapsulates alpelisib. Alpelisib inhibits the PI3K-Akt-mTOR signaling pathway in tumor cells and disrupts osteoclast activity. Following modification with ICG, the AMMD surface displays a strong LSPR effect, indicating potential for PTT and PAI. ICG was specifically selected for use with spherical gold nanoparticles. The choice aimed to prevent light quenching caused by spectral emission overlap with gold nanorods (Ma et al., 2021).

##### 2.1.2.2 Copper nanoparticles

Among emerging nanomaterials for PTT, copper (Cu) nanoparticles have attracted attention due to their biodegradability, moderate cellular tolerance, and surface plasmon resonance near the NIR-I boundary (550–600 nm). Additionally, copper chalcogenides (Cu_2-x_E; E = S, Se, Te) have been widely investigated for NIR-triggered applications, including PAI and thermal ablation. Uniquely, Cuprous sulfide featuring Cu-deficient stoichiometries (Cu_2−x_S) demonstrates stoichiometry-dependent LSPR in the NIR, driven by cation vacancy-induced free holes rather than electron oscillations (Tai et al., 2018; Dong et al., 2020).

Unlike the typical LSPR. Hollow copper sulfide nanoparticles (HCuS NPs) exhibit NIR absorption mainly from dd transitions, which is independent of particle size or shape (Tong et al., 2023). Another material, copper-molybdenum sulfide (CMS), contains polyvalent metal ions (Cu, Mo) with strong NIR absorption and suits for antitumor applications (Yao et al., 2022).

Copper-based nanomaterials exhibit significant photothermal activity under NIR laser irradiation. Owing to their nanoscale dimensions, copper sulfide nanoparticles efficiently accumulate in tumor tissues via enhanced permeability and retention effects. Subsequent NIR activation induces tumor ablation, minimizing damage to adjacent healthy tissues (Lan et al., 2021). Beyond the photothermal effect, some materials provide alternative therapeutic mechanisms. Copper sulfide nanoparticles release copper ions within the acidic tumor milieu. The released copper ions generate reactive oxygen species (ROS) via a Fenton-like reaction involving endogenous hydrogen peroxide (Lan et al., 2021). Furthermore, CMS effectively generates cytotoxic superoxide anions (O2⋅−) upon 808 nm laser irradiation (Yao et al., 2022). Advanced platforms enable targeted and combination approaches. For instance, HCuS NPs integrated with pHLIP actively target acidic tumor regions, a capability confirmed through *in vivo* imaging (Tong et al., 2023). Radiation exposure involving the nanoparticles triggers PTT and immunogenic cell death. The consequent hyperthermia offers further utility: it can melt lauric acid to release the stress granule inhibitor ISRIB. ISRIB subsequently sensitizes cells to PTT (Tong et al., 2023).

##### 2.1.2.3 Carbon nanoparticles

Carbon-based nanomaterials, particularly carbon nanotubes (CNTs), have been extensively explored in PTT due to their favorable physicochemical properties. Structurally composed of one or more concentric layers of carbon atoms in hexagonal arrays, CNTs exhibit mechanical robustness, thermal stability, and efficient NIR photothermal conversion. Multiwalled CNTs are especially effective in tumor ablation via localized hyperthermia. Although their native hydrophobicity hinders aqueous dispersion, functionalization with surfactants, proteins, polysaccharides, or polyethylene glycol (PEG) enhances solubility, biocompatibility, and therapeutic versatility (Liu et al., 2025).

Carbon nanomaterials have unique physical and chemical properties that are valuable for therapeutic purposes. Their high surface area facilitates bulk loading of aromatic drugs. In addition, their surfaces are easily modified by various biofunctional groups, including hydroxyl, epoxide, carboxyl and amino groups. Such functionalization improves their aqueous solubility, stability in physiological solutions, and the enhanced drug-loading capacity (Lu et al., 2014). Graphene nanoparticles also exhibit photothermal anticancer activity *in vitro*. Such effects include induction of oxidative stress and mitochondrial damage leading to apoptosis and necrosis of tumor cells. Studies have shown that graphene nanoparticles have higher photothermal anticancer efficiency than CNTs due to their smaller size and better dispersion (Markovic et al., 2011). Qian et al. prepared 6–8 nm hollow carbon nanodots (HCCDs) possessing a crystalline core and hydrophilic surface. The HCCDs exhibit strong photoacoustic and photothermal properties, as well as tunable fluorescence. In mice with U87 gliomas, HCCDs specifically concentrated within brain tumors. Their accumulation facilitates dual-mode imaging guidance for PTT, leading to effective anti-tumor results while minimizing damage to normal tissue (Qian et al., 2018).

##### 2.1.2.4 Silicon nanoparticles

Silicon-based nanomaterials constitute a key group of photothermal agents. Their appeal arises from favorable biocompatibility, biodegradability, and abundant availability. Porous silicon nanoparticles, in particular, are widely employed in biomedical applications. Typically produced through electrochemical etching of p-type Si (100) wafers, the nanoparticles possess tunable band gap energies ranging from 1.12 to 2.5 eV. Quantum confinement and an irregular pore distribution account for this tunable property. These structural attributes promote efficient photothermal conversion. The conversion is mediated by electron-hole recombination under laser excitation, highlighting their potential in PTT (Yu et al., 2017).

Atomically thin silicon quantum sheets (Si QS), measuring approximately 14.0 nm in transverse dimension and 1.6 nm in thickness, exhibit high mass extinction coefficients (27.5 L g^−1^ cm^−1^) and remarkable photothermal conversion efficiencies (47.2%) at 808 nm, which are reported to outperform other reported 2D mono-elemental materials (Xenes) (Miao et al., 2021). Si QSs have low toxicity and well-balanced stability, and can degrade to non-toxic orthosilicic acid *in vivo*. Their ultrasmall size and layered structure enable them to cross the blood-brain tumor barrier and efficiently accumulate in glioma tissues through the enhanced penetration and retention effect, without the need for specific targeting modifications (Miao et al., 2021).

Mesoporous silica nanoparticles (MSNs) serve as multifunctional platforms. MSN surfaces possess abundant silanol groups suitable for functionalization, enabling controlled drug delivery (Wang et al., 2014). For instance, Wang et al. developed TsGMSN using MSN technology. TsGMSN incorporates semi-graphitized carbon (sGC) hotspots deposited on inner pore walls. The arrangement ensures direct contact between loaded hydrophobic drugs, like DOX, and the sGC hotspots. Consequently, alterations in pH and NIR radiation can effectively trigger drug release. Locating hydrophobic sGC on a hydrophilic silica wall establishes TsGMSN as an effective therapeutic carrier. Despite moderate sGC content (7.2%) and graphitization degree, a regular distribution of the hotspots provides the nanoparticles with excellent photothermal efficiency (Wang et al., 2014).

##### 2.1.2.5 Iron oxide nanoparticles

Iron oxide nanoparticles, particularly those with a magnetite crystal phase, have been identified as highly efficient for PTT, with magnetite nanocubes and magnetite magnetosomes demonstrating substantial heating in the NIR spectrum. The heating power of these materials can exceed 1–10 kW gFe^−1^, surpassing typical magnetic hyperthermia thresholds (Van de Walle et al., 2023).

Supramagnetic iron oxide (SPIO) nanoparticles serve as a prominent example. Considered a standard magnetic particle imaging tracer, SPIO is highly regarded for non-radioactivity, extended stability, and biocompatibility, alongside having FDA clinical approval (Huang X. et al., 2023). Furthermore, iron oxide nanoparticles allow integration into sophisticated structures. Wang et al., for example, utilized macrophages for delivering MFe_3_O_4_-Cy5.5 nanoparticles. Macrophage-mediated delivery enables multimodal fluorescence, photoacoustic, and magnetic resonance imaging (MRI). The approach provides advantageous probing depth plus a high signal-to-noise ratio. The combination of imaging modalities helps distinguish brain tumors from normal tissue and offers precise guidance during glioma resection (Wang S. et al., 2021).

Some inorganic nanomaterials containing selenium and vanadium are also worth noting. Dai et al. focused on Bi_2_Se_3_ nanodisks possessing remarkable ROS scavenging via multienzymatic activity. This characteristic counteracted inflammation typically associated with PTT. Furthermore, these nanodisks exhibited computed tomography capabilities, enabling integrated diagnosis and treatment functions (Dai et al., 2024). Guo et al. prepared TA-VOx nanobelts. The formation involved tannic acid (TA), whose abundant catechol structures provide chelating sites for metal ions, facilitating a self-assembled metal-polyphenol network. Furthermore, TA’s reduction capacity reduces V^5+^ to V^4+^ ions, partly enhancing the material’s NIR absorption ability (Guo et al., 2022).

The information involving the classification, parameters and functionalization of the above mentioned nanomaterials for PTT applied to neural tumors is shown in Table 1.

**TABLE 1:** Summary of engineered nanomaterials for PTT targeting neural tumors.

Classification	Specific nanomaterial	Size (nm)	Key modifications	PTT parameters (λ, power density)	Photothermal conversion efficiency (%)	Treated disease	Cellular models/animal models	Ref.
Liposomes	I&T@LipA	104.65 ± 7.45 nm	IR780 and Drug Temozolomide loading	808 nm, 2.0 W/cm^2^	45.4%	Gliomas	Orthotopic glioma mouse model (nude BALB/c mice)	Zeng et al. (2023)
Liposome	vitexin/ICG liposome	107.33 ± 1.03 nm	vitexin and ICG loading	N/S	N/S	Gliomas	U251 cells	Cao et al. (2023a)
Liposome	Lipo@HRP&ABTS	∼100 nm	HRP and ABTS loading	808 nm, ∼0.8 W/cm^2^	N/S	Gliomas	U87MG cells	Chen et al. (2017)
Polydopamine	FLDIPs	250 ± 1 nm	DOX and ICG loading	808 nm, 2 W/cm^2^	51.4%	Gliomas	C6 glioma cells	Dube et al. (2021)
Polydopamine	^211^At-PDA-FAPI	160–180 nm	Radiolabeling with ^211^At	808 nm, 1.0 W cm^−2^	35.2%	Gliomas	U87MG cells	Li et al. (2024a)
Polydopamine	Pep-1@PDA-TMZ NPs	140 nm	Pep-1 conjugation	808 nm, 1 W/cm^2^	N/S	Gliomas	U87 cells	Wu et al. (2023)
Peptide	PINPs	348 nm	ICG loading	NIR-808 laser, 2 W/cm^2^	N/S	Gliomas	C6 cells	Dube and Panda (2023)
Peptide	HSA-ICG-MB NPs	75.0 nm (TEM), ∼110.0 nm (DLS)	crosslinked by glutaraldehyde	808 nm, 1.0 W/cm^2^ (*in vitro*)	N/S	Gliomas	C6 glioma cells	Hu et al. (2018)
Peptide	BLIPO-ICG	104 ± 3 nm	glioma cell membrane proteins embedding	808 nm, 1 W/cm^2^	N/S	Gliomas	C6 glioma cells	Jia et al. (2019)
Peptide	ICG-PEP-c (RGD)fk (IPR)	20.74 ± 6.70 nm	Glutathione-reactive self-assembling PEP as skeleton	808 nm, 1 W/cm^2^	37.68%	Gliomas	U87MG cells	Song et al. (2023a)
Peptide	ICG-CSFNPs	120.1 nm	ICG loading	808 nm, 1 W/cm^2^	N/S	Gliomas	C6 glioma cells	ZhuGe et al. (2019)
Dye-containing nanoparticles	C12-TPAE-AL	110.4 and 108.59 nm (TEM and DLS)	ApoE peptide decoration	1,064 nm, 1 W/cm^2^	62.4%	Gliomas	U87 cells	Huang et al. (2024)
Apoptotic Bodies	DI/Abs	1 µm	DOX and ICG loading	808 nm, 1.0 W/cm^2^	N/S	Gliomas	C6 glioma cells	Liu et al. (2023b)
Exosomes	EE@Fs-NPs	110 nm	Encapsulating Fs with microglia-exosomes which express anti-LAG3	808 nm, 1 W cm^−2^	N/S	Gliomas	GL261 cells	Lin et al. (2023)
Polymer	iRGD-PEG-PLGA/A1094T/TMZ (PA1094T) NPs	≈128 nm	iRGD peptide conjugation	1,064 nm, 1.2 W cm^−2^	31%	Gliomas	GL261 cells	Li et al. (2025)
Polymer	PDTT-253 NPs	220.4 nm	embedding D1, D2, π1, and A2 into the backbone	808 nm, 800 mW/cm^2^ (*in vitro*)	85.1%	Gliomas	U87 cells	Su et al. (2025)
Gold	ASQ-PGEA	150–250 nm	pDNA and DOX loading	808 nm, 2 W/cm^2^	N/S	Gliomas	C6 glioma cells	Duan et al. (2017)
Gold	Bt@Au-NPs	69.44 ± 1.94 nm	Biotin conjugation	360 nm (UV irradiation),N/S	N/S	Gliomas	C6 glioma cells	He et al. (2021)
Gold	AuNSs-RGD-C≡C-siRNA (ARCR)	∼150 nm	Functionalized with RGD peptides and siRNA	808 nm, 0.5 W/cm^2^ (*in vitro*)	50.26%	Gliomas	U87 cells	Hu et al. (2024)
Gold	HGNPs	95–135 nm	PVP-coated	660nm, 1.5 W/cm^2^	N/S	Gliomas	C6 rat glioma cells	Liu et al. (2024b)
Gold	Au@MOF@MS-ICG-dYNH-PAA (AMMD)	123.7 ± 8.6 nm	dYNH peptide conjugation	808 nm, 1.2 W/cm^2^	N/S	Spinal metastases from *in situ* tumors	A549 cells	Ma et al. (2021)
Copper	Tf-DSF/CuS	171.9 ± 1.9 nm	Transferrin modification	808 nm, 2 W/cm^2^	37.62%	Gliomas	C6 cell	Lan et al. (2021)
Copper	IL@H-PP	222.1 ± 1.3 nm	PEGylation	808 nm, 2 W/cm^2^	N/S	Brain metastases	4T1 cells	Tong et al. (2023)
Copper	CMS/PEG-DOX-M	<100 nm	PEGylation	808 nm, 1 W·cm^−2^	N/S	Gliomas	U87 MG	Yao et al. (2022)
Carbon	PL-PEG-GONRs	77.01 ± 0.38 nm	PEGylation	808 nm, 2 W/cm^2^	N/S	Gliomas	U87 glioma cells	Lu et al. (2014)
Carbon	graphene nanoparticles	50 nm (single-layer)	PVP-coated	808 nm, 2 W/cm^2^	N/S	Gliomas	U251 human glioma cells	Markovic et al. (2011)
Carbon	HCCDs	6–8 nm	hydrophilic surface groups	808 nm, 2 W cm^2^ (*in vitro*)	42.3%	Gliomas	U87 glioblastoma cells	Qian et al. (2018)
Silicon	2D silicon quantum sheets (Si QSs)	14.0 nm (lateral size), 1.6 nm (thickness)	PVP-capped	808 nm, 2 W (*in vitro*)	47.2%	Gliomas	C6 cells	Miao et al. (2021)
Silicon	TsGMSN	115 ± 20 nm	DOX loading	808 nm, 6 W cm^−^	N/S	Gliomas	glioma U251 cells	Wang et al. (2014)
Iron oxide	CCM-SPIO	≈87.4 nm	CCM coating	785 nm, 0.8 W cm^−2^	N/S	Gliomas	bEnd.3 cells	Huang et al. (2023b)
Iron oxide	MFe3O4-Cy5.5	190 nm	Cy5.5 conjugation	808 nm, 1 W/cm^2^	49.14%	Gliomas	C6 glioma cells	Wang et al. (2021a)
Bi	Bi_2_Se_3_ nanodisks	N/S	PVP coating	808 nm, 2.0 W/cm^2^	43.9%	Gliomas	GL261 cells	Dai et al. (2024)

### 2.2 Neurodegenerative diseases

#### 2.2.1 Organic nanoplatform

##### 2.2.1.1 Polydopamine nanoparticles

PDA NPs have demonstrated multifunctionality relevant to the treatment of neurodegenerative diseases. Chen et al. focused on their promising photothermal conversion capabilities. In addition, they scavenge Aβ-Cu^2+^-induced intracellular ROS, thereby reducing the relevant cellular damage (Chen et al., 2023). Based on the properties of PDAs, Liu et al. developed PDA-CQDRBC, a composite system combining nitrogen-doped carbon quantum dots (CQDs) with erythrocyte membrane-coated PDAs. Nitrogen doping enhances the absorption/emission and photothermal efficiencies of CQDs, and PDA-CQDRBC exhibits significant Cu^2+^ binding ability. It retarded Aβ nucleation and inhibited Cu^2+^-induced Aβ aggregation, which was confirmed by the reduction of thioflavin T fluorescence. Although Cu^2+^ normally enhances Aβ fibrosis and toxicity, co-incubation with PDA-CQDRBC significantly increased cell viability in a concentration-dependent manner. This protective effect stems from the ability of nanomaterials to resist aggregation and reduce basal ROS levels (Liu et al., 2024a).

##### 2.2.1.2 Other polymeric nanoparticles

Chen et al. developed BDPHPC, which exhibits thermally controlled properties governed by its lowest critical solvation temperature (LCST). Below LCST, it exhibits a hydrophilic, randomly curled state in which π-π stacked, rotationally restricted boron-dipyrrole methylene (BDP) molecules efficiently dissipate absorbed NIR photons in the form of heat. The photothermal effect can be controlled by both concentration and laser power. Above LCST, the BDPHPC transforms into a dehydrated fiber conformation. At this point, the BDP molecule becomes a free-spinning monomer with high photoluminescence, which enhances its specificity as an NIR fluorescent probe against Aβ species. Such a reversible phase transition can burst and temporarily reverse Aβ_42_ peptide fibrillation (Chen et al., 2022).

#### 2.2.2 Inorganic nanoplatform

##### 2.2.2.1 Carbon nanoparticles

Carbon-based nanomaterials offer a variety of strategies for light-mediated AD therapies targeting Aβ pathology. GO-ThS utilizes the ability of graphene oxide to locally generate heat under NIR laser irradiation, which contributes to selective Aβ protofibril dissociation. GO-ThS also exerts a protective effect against Aβ-related cytotoxicity under NIR light irradiation (Li et al., 2012). Another approach is to employ KD8@N-MCN based on nitrogen-doped mesoporous carbon nanospheres (N-MCN), which can be used as NIR-II photothermite. Their structure is characterized by dispersed sp2 hybridized pore walls that promote light absorption and photothermal conversion. In addition, graphitic nitrogen dopants contribute excess electrons that enhance the light absorption properties. N-MCN uniquely exhibit intrinsic superoxide dismutase (SOD) and catalase activity. The enzymatic function allows them to counteract the oxidative damage associated with AD (Ma et al., 2020). L-CNDs have a unique dual function: they bind Aβ aggregates through hydrophobic interactions and π-π stacking. Simultaneously, L-CNDs have a strong 630 nm absorption and good fluorescence properties. They have minimal toxicity to PC12 and HT22 cells, while reducing Aβ-induced cellular damage (Shao et al., 2025). Yan et al. also developed YCDS-CE6, which employs yellow carbon dots as the NIR response core. Under combined PDT and PTT, YCDS-CE6 effectively inhibited amyloid fibrillation and completely disintegrated mature fibrils. It also inhibited the conformational transition of the protein from α-helical to β-folded structure. In a cellular model, Aβ_42_ aggregate adhesion was reduced and Aβ_42_-induced neurotoxicity was attenuated after treatment with YCDS-CE6 (Yan et al., 2022).

##### 2.2.2.2 Molybdenum nanoparticles

Molybdenum-based nanomaterials, encompassing molybdenum disulfide, molybdenum oxides, molybdates, and Mo-based polyoxometalates, have generated significant interest. Tunable valence states and multifunctional properties are key reasons for investigation. High NIR photothermal conversion efficiency, substantial conductivity, large surface area, and favorable biocompatibility promote integration into biomedical applications. Particularly PTT (Liao et al., 2023).

Qi et al. investigated molybdenum disulfide quantum dots (MoS_2_ QDs). Macrophage membranes were employed for biomimetic modification, aiming to improve QD performance. Exploiting macrophage immunological characteristics potentially enhances evasion of the reticuloendothelial system and prolongs circulation time for the modified QDs. Additionally, the inherent MoS_2_ material possesses anti-Aβ_1-42_ aggregation effects. It also acts as an effective nano-enzymatic scavenger of free radicals. Liposomes provided an alternative biomimetic modification for MoS_2_ QDs, augmenting the material’s anti-aggregation and scavenging capabilities. The Liposome coating effectively reduces recognition and phagocytosis by macrophages, facilitating escape from immune clearance (Qi et al., 2024).

##### 2.2.2.3 Other inorganic nanoparticles

There are other inorganic nanomaterials that are being designed for therapeutic intervention in neurodegenerative diseases. For instance, Zhou et al. developed hollow ruthenium nanoparticles loaded with nerve growth factor (NGF). This design enabled controlled, NIR-triggered delivery and improved BBB permeability. The system inhibited tau hyperphosphorylation and aggregation. In AD mice, it reduced oxidative stress, repaired neurological damage, and improved memory (Zhou et al., 2020).

Black phosphorus (BP), a 2D nanomaterial, demonstrates potent ROS scavenging abilities. A BP-based composite was shown to reduce mitochondrial oxidative stress and α-synuclein accumulation (Cheng et al., 2023). BP also possesses a tunable energy bandgap for broad spectrum absorption. It degrades into biocompatible oxides, ensuring low toxicity (Xiong et al., 2020).

Gold nanorods (GNRs) were employed to construct a dual-function system (GAS) for Aβ aggregation. Without NIR irradiation, GAS serves as a visual detector. Upon NIR exposure, GNR-induced hyperthermia disrupts Aβ protofibrils and activates thermophilic alkaline phosphatase ST0779. The activated system then degrades Aβ, inhibits further aggregation, and reduces Aβ-mediated peroxidase activity, thereby mitigating toxicity (Liu D. et al., 2019). Sudhakar et al. attributed the photothermal effect of Silver Triangular Nanoprisms (AgTNPs) to hyperthermia induced by in-plane dipole resonance. Similarly, NIR-irradiated AgTNPs diminished fibril content and enhanced cell viability (Sudhakar and Mani, 2019).

Prussian blue nanoparticles (PBNPs) exhibit intrinsic enzyme-like activities. One PBNP system, coated with erythrocyte membranes, inhibits and depolymerizes Aβ aggregates via photothermal effects. It also mitigates ROS, eliminates plaques, and repairs memory in mouse models (Li L. et al., 2024). Another was modified with peptides to target Aβ across the BBB (Song X. et al., 2023). In a different approach, a nanosystem combined CQDs, cerium oxide (Ce), and macrophage membranes. The membrane exhibited anti-inflammatory properties, while ultra-small Ce particles grown on it scavenged ROS to mitigate oxidative stress, thereby enhancing the ability of CQDs to inhibit Aβ aggregation (Chi et al., 2024).

#### 2.2.3 Special composite nanoplatform

Composite nanomaterials offer multifaceted therapeutic avenues. Ge et al. developed U-CNCoP, which combines graphitic carbon nitride (g-C_3_N_4_) with cobalt phosphide (CoP) cocatalyst. The incorporation of CoP accelerated the segregation and transfer of photogenerated electrons within g-C_3_N_4_, thereby enhancing photocatalytic activity. The material exhibited strong anti-oxidative stress mitigation and suppressed Aβ aggregation. In addition, U-CNCoP exerted extensive neuroprotective effects and significantly enhanced cognitive function in an AD mouse model (Ge et al., 2023). Another approach by Ge et al. utilized KLVFFAu-CeO_2_ (K-CAC) nanocomposites. In this structure, CeO_2_ provides antioxidant ROS scavenging capacity through its Ce^3+^/Ce^4+^ redox cycle, thus preventing cellular damage. Au NRs were used as carriers for adsorption of the Aβ-targeting inhibitory peptide KLVFF. Tip modification of gold nanorods with CeO_2_ improved the photothermal conversion efficiency. At the same time, the gold nanorod plasma extended the CeO_2_ photocatalysis into the NIR spectrum via hot electron injection, which enhanced the redox properties of cerium dioxide. K-CAC exhibited enhanced BBB permeability. KLVFF peptide component promotes Aβ targeting, inhibits the aggregation of monomers, and contributes to minimizing the damage to normal tissues (Ge et al., 2022).

The information involving the classification, parameters and functionalization of the above mentioned nanomaterials for PTT applied to neurodegenerative diseases is shown in Table 2.

**TABLE 2:** Summary of engineered nanomaterials for PTT targeting neurodegenerative diseases.

Classification	Specific nanomaterial	Size (nm)	Key modifications	PTT parameters (λ, power density)	Photothermal conversion efficiency (%)	Treated disease	Cellular models/animal models	Ref.
Polydopamine	polydopamine nanoparticles	103.15 ± 4.64 nm	N/S	808 nm, 2 W·cm^−2^	22.3%	Alzheimer’s disease	SH-SY5Y cells	Chen et al. (2023)
Polydopamine	PDA-CQD/RBC	169.9 ± 2.9 nm	Erythrocyte membrane coating	808 nm, 2 W/cm^2^	34.64%	Alzheimer’s disease	SH-SY5Y cells	Liu et al. (2024a)
Carbon	GO-ThS	∼1.5 nm	ThS-modified	808 nm, 1 W/cm^2^	N/S	Alzheimer’s disease	PC12 cells	Li et al. (2012)
Carbon	KD8@N-MCNs	100 nm	KLVFFAED peptides	1,064 nm, 0.8 W/cm^2^	45.86%	Alzheimer’s disease	PC-12 cells	Ma et al. (2020)
Carbon	L-CNDs	∼8.5 nm	N/S	808 nm, 0.6 W/cm^2^	68.25%	Alzheimer’s disease	PC-12 cells	Shao et al. (2025)
Carbon	yCDs-Ce6	37.2 nm	Coassembling photosensitizers and yellow fluorescent carbon dots	808 nm, 1.5 W/cm^2^	54.2%	Alzheimer’s disease	PC-12 cells	Yan et al. (2022)
Molybdenum	MoS2 QDs/MM	125.62 ± 4.132 nm	Modified by macrophage membrane (MM)	808 nm, 2 W cm^2^	N/S	Alzheimer’s disease	SH-SY5Y cells	Qi et al. (2024)
Molybdenum	MoS2 QDs/lipid-Cur	208.95 ± 8.342 nm	Functionalized with lipids	808 nm, 2 W/cm^2^	N/S	Alzheimer’s disease	SH-SY5Y cells	Qi et al. (2023)
Ruthenium	NGF-PCM@Ru NPs	164.2 nm	NGF loading	808 nm, 1 W/cm^2^	N/S	Alzheimer’s disease	SH-SY5Y cells	Zhou et al. (2020)
Black phosphorus	Lf-BP-Pae	203.1 nm	Lf conjugation	808 nm, 0.5 W/cm^2^	33.4%	Parkinson’s disease	SH-SY5Y cells	Xiong et al. (2020)
Gold	GNRS-APH-scFv (GAS)	30 nm (length),8.3 nm (width)	Conjugated with thermophilic acylpeptide hydrolase	808 nm, 2 W cm^−2^	N/S	Alzheimer’s disease	SH-SY5Y cells	Liu et al. (2019a)
Silver	AgTNPs	70 ± 8 nm	PVP-capped	800 nm, 200 mW/mm^2^	N/S	Alzheimer’s disease	SH-SY5Y cells	Sudhakar and Mani (2019)
Prussian blue	PB/RBC	73.6 ± 2.5 nm	Encapsulated with RBC membranes	808 nm, 1.0 W/cm^2^	46.2%	Alzheimer’s disease	SH-SY5Y cells	Li et al. (2024b)
Prussian blue	PBK NPs	163.5 ± 10.0 nm	PEI coating	808 nm, 1 W/cm^2^	48.2%	Alzheimer’s disease	PC12 cells	Song et al. (2023b)
Ce	CQD-Ce-RAW	195.4 ± 4.2 nm	Modified by macrophage membrane	808 nm, 2 W/cm^2^	37%	Alzheimer’s disease	SH-SY5Y cells	Chi et al. (2024)
Polymer	BDP-HPC	94–152 nm	a molecular rotor-based boron dipyrromethene photosensitizer conjugation	680 nm, 1 W cm^−2^	78.1%	Alzheimer’s disease	PC12 cells	Chen et al. (2022)
Special composite nanoplatform	UCNP@g-C3N4/CoP (U-CN/CoP)	∼80 nm	CoP cocatalyst	980 nm, 1 W/cm^2^ (10 min)	36.8%	Alzheimer’s disease	PC12 cells	Ge et al. (2023)
Special composite nanoplatform	KLVFF@Au-CeO2 (K-CAC)	∼100 nm	CeO2 deposition	808 nm, 0.5 W/cm^2^	41.5%	Alzheimer’s disease	PC12 cells	Ge et al. (2022)

### 2.3 Comparative analysis of organic and inorganic nanoplatforms for neuro-applications

The successful application of photothermal therapy in neurological disorders hinges on the careful selection of nanomaterials. While both organic and inorganic nanoplatforms have shown promise, their inherent differences significantly influence their suitability for specific neuro-applications. This section provides a comparative analysis of these two classes of nanomaterials. We will delve into their biocompatibility and how they interact with biological systems, the scalability and cost-effectiveness of their production, and the regulatory hurdles that must be overcome for clinical use.

#### 2.3.1 Biocompatibility and biodegradation

Organic nanomaterials generally present favorable safety profiles for biomedical applications. Many polymeric nanostructures, for instance, are inherently biodegradable (Bhargav et al., 2020). Specific examples include polycaprolactone, a low-cost, FDA-approved bioresorbable polyester (Chen et al., 2020), and polydopamine, which offers excellent biocompatibility (Dube et al., 2021). Lipid-based nanoparticles and nanogels are also valued for high biodegradability and can be cleared from the body (Bhargav et al., 2020; Vashist et al., 2023). The photosensitizer ICG shows low biotoxicity, binding to plasma proteins for rapid liver excretion (Cao X. et al., 2023). ICG molecules can also decompose during photothermal heating (Hu et al., 2018).

Conversely, the biological fate of inorganic nanomaterials presents challenges. Many are non-degradable and can accumulate, raising toxicity concerns (Lee et al., 2024). The physiological compatibility of single-walled carbon nanotubes remains debated (Sagar and Nair, 2018), while the non-degradable nature of gold nanoparticles prompts concerns about cumulative toxicity (Turkmen Koc et al., 2024). Nevertheless, certain inorganic systems, including zirconium-based MOFs and Bi_2_Se_3_ nanodisks, have demonstrated low toxicity (Bybordi et al., 2024; Dai et al., 2024). Furthermore, research indicates cellular degradation of AuNPs is possible, depending on particle size (Dube et al., 2022).

To improve systemic tolerance, nanocarriers are frequently engineered to evade the mononuclear phagocyte system (MPS) (Joseph and Nance, 2022). Surface modification is a key strategy; coating nanostructures with polymers like PEG can mitigate toxicity and limit MPS clearance (Vashist et al., 2023; Turkmen Koc et al., 2024). An advanced technique involves camouflaging nanoprobes with biomimetic membranes, such as those from platelets. This approach boosts biocompatibility and helps the nanostructures elude immune detection (Geng et al., 2020; Huang X. et al., 2023).

#### 2.3.2 Manufacturing scalability and cost-effectiveness

The synthesis of organic photothermal agents has evolved beyond the difficult processes of traditional methods (Lee et al., 2024). Modern strategies like self-assembly offer a simpler route, bypassing complex synthesis and processing stages (Dube and Panda, 2023). Some fluorescent nanoparticles are now fabricated through a straightforward, one-step green method that requires no organic solvents (Dube et al., 2021).

Manufacturing technology shows similar progress. Microfluidics enables precise control over nanoparticle properties, a clear improvement over conventional batch production (Rabiei et al., 2020). This technique allows for precise regulation of liposome size and distribution, yielding high drug-loading efficiency and stability (Cao X. et al., 2023). Cost-effectiveness is improved by using peptide ligands, which are cheaper to produce than antibodies (Rabiei et al., 2020). Additionally, biomimetic nanoprobes derived from abundant platelets hold immense potential for clinical translation (Geng et al., 2020).

While inorganic agents can be costly and difficult to reproduce (Lee et al., 2024), innovative methods now permit scalable, economical production. For example, TA-VOx nanobranches are synthesized via a simple, one-step self-assembly (Guo et al., 2022). Gold nanorose creation has been refined into a fast, green aqueous process (Dube et al., 2022). Scalable techniques for metal nanoparticles include chemical reduction and microwave-assisted synthesis (Singh et al., 2025). Despite such progress, significant financial and technical hurdles still impede the clinical translation of gold nanoparticles, limiting widespread commercial use (Turkmen Koc et al., 2024).

#### 2.3.3 Regulatory hurdles and translational potential

The clinical translation of nanomedicines is impeded by an inadequate systematic exploration of nano-bio interfaces. Upon entering a biological system, nanoparticles acquire a biomolecular corona that profoundly impacts the pharmacokinetic profile, yet research often overlooks how nanocarriers affect vital cellular functions like mitochondrial metabolism (Sagar and Nair, 2018; Bhargav et al., 2020). Progress is further stalled by a lack of uniform methodologies for evaluating toxicity, efficacy, brain uptake, and NIR dosage control (Sagar and Nair, 2018; Bhargav et al., 2020; Joseph and Nance, 2022).

Significant safety considerations also arise. Even targeted nanoformulations exhibit substantial accumulation in the liver, spleen, and kidneys (Rabiei et al., 2020). The long-term *in vivo* fate of materials like gold nanoparticles remains ambiguous, and non-degradable delivery systems necessitate subsequent surgical removal (Bybordi et al., 2024; Turkmen Koc et al., 2024). For brain therapies, cranial obstruction of NIR light often requires invasive craniotomy (Guo et al., 2022).

Furthermore, preclinical models that fail to recapitulate clinical complexities, such as tumor heterogeneity, can produce misleading outcomes (Bhargav et al., 2020). Testing across multiple animal models, including large animals, is therefore essential before human studies (Joseph and Nance, 2022). Despite these challenges, some nanomedicines have entered early-phase clinical trials for brain cancer (Bhargav et al., 2020). Future advancements depend on designing stimuli-responsive platforms for remote release and personalized systems, like those using patient-specific cancer cell membranes, to address tumor heterogeneity (Rabiei et al., 2020; Huang X. et al., 2023).

## 3 Applications of PTT in neural tumors and neurodegenerative diseases

### 3.1 PTT for neural tumors

#### 3.1.1 PTT and glioma

Gliomas are the most common tumors in the nervous system, accounting for nearly half of all brain tumors, with an annual incidence of approximately 30–80 cases per million people (Li T. et al., 2022). A common approach for treating glioma is PTT, which utilizes the penetrating ability of NIR light. In this process, photosensitizers with high photothermal conversion efficiency absorb light and transform it into thermal energy, achieving tumor ablation through heat (Liu D. et al., 2023). To improve precision, nanoparticles integrated with PAI and MRI allow for accurate localization of tumor cells. This capability enables targeted thermal therapy while minimizing side effects (Guo et al., 2018; Tao et al., 2024). Advanced delivery systems, such as photothermally active micromotors, can generate a temperature gradient under continuous illumination. This creates a thermophoretic force that propels the micromotor forward and can trigger dopamine polymerization in the brain, enhancing the regionalization of PTT (Li Z. et al., 2023). These micromotors can also be loaded with chemotherapeutic agents to achieve concurrent anti-tumor effects (Li H. et al., 2023).

While PTT can destroy glioma cells independently, its therapeutic efficacy is constrained by factors like cancer metastasis, patient variability, nanomaterial biotoxicity, and inflammation (Hildebrandt et al., 2002; Cheng et al., 2014; Melamed et al., 2015; Liu Y. et al., 2021; Overchuk et al., 2023). Consequently, research indicates that integrating PTT with other treatments enhances its effectiveness, reduces adverse effects, and improves the overall patient experience (Cheng et al., 2014; Liu et al., 2019b; Xie et al., 2020; Xu and Liang, 2020; Huang et al., 2022; Qiao et al., 2022; Zhang et al., 2022a; Overchuk et al., 2023).

PTT demonstrates significant synergy with chemotherapy through several mechanisms. For instance, mild photothermal effects (40°C–44°C) can increase blood flow, improving drug delivery to the tumor (Cao Y. et al., 2023). Other strategies achieve controlled chemotherapy release. One method uses NIR light to trigger the separation of a gatekeeper molecule, releasing DOX and p53 for concurrent gene therapy (Duan et al., 2017). Another employs a magnetic field to guide nanomaterials, with subsequent DOX release prompted by PTT-induced heat and high tumoral ROS (Jiang et al., 2024). Furthermore, PTT-induced hyperthermia enhances the cellular uptake of nanoparticles, leading to severe lysosomal disruption (Huang et al., 2019). The elevated temperatures also directly increase the cytotoxicity of chemotherapeutic agents (Dong et al., 2016).

A key advantage of PTT is its ability to modulate the tumor microenvironment, thereby sensitizing gliomas to both chemotherapy and radiotherapy. Specifically, PTT can enhance blood flow within tumors and alleviate hypoxia (Li J. et al., 2023). Studies confirm that combining PTT with chemotherapy reduces the expression of hypoxia-inducible factor-1 (Zeng et al., 2023). This approach has been shown to lessen tumor hypoxia and inhibit glioma growth *in situ* (Zhou et al., 2024). The sensitizing effect also benefits radiotherapy, as experiments reveal that combining PTT with radiation yields a greater destructive impact on cells than radiotherapy alone (Kargar et al., 2018). An innovative strategy combined PTT with hyperbaric oxygen and chemotherapy to overcome temozolomide (TMZ) resistance and increase the chemosensitivity of glioma stem cells (Zeng et al., 2019).

PTT can also be leveraged to initiate a potent anti-tumor immune response, a strategy known as photothermal immunotherapy. The photothermal ablation of cancer cells induces immunogenic cell death (ICD), releasing tumor-associated antigens (TAAs) and other molecular patterns that activate the immune system (Xu and Liang, 2020; Sun et al., 2022). The resulting systemic response can target residual or metastatic tumors and establish immune memory to prevent recurrence (Xu and Liang, 2020; Huang et al., 2021). This process effectively transforms immunologically “cold” tumors into “hot” ones, making them more susceptible to immune checkpoint blockers (Xie et al., 2020; Zhao et al., 2022). Supporting this, one study found that combining PTT with an anti-PD-1 antibody produced the strongest inhibition of tumor regeneration (Nie et al., 2023). Beyond ICD, PTT has also been shown to reprogram tumor-associated macrophages from a tumor-promoting to a tumor-suppressing phenotype, further boosting immunotherapy’s effectiveness (Overchuk et al., 2023).

#### 3.1.2 PTT and peripheral nerve sheath tumors

Peripheral nerve sheath tumors are rare, aggressive sarcomas with a generally poor prognosis. Given that these tumors are resistant to traditional chemotherapy and lack effective targeted therapies, current treatment options remain challenging (Sweeney et al., 2016). PTT can directly eliminate tumors via photothermal ablation. It may also decrease the expression of phosphorylated extracellular regulated protein kinases (ERK). In conjunction with mitogen-extracellular kinase inhibitors, PTT synergistically influences the oncogene Ras signaling pathway. This occurs by impeding ERK activation, thereby promoting cell apoptosis and necrosis (Sweeney et al., 2016). Furthermore, the high temperatures generated during PTT can induce endoplasmic reticulum stress (ERS) in tumor cells. ERS consequently triggers endogenous apoptosis (Gu et al., 2022). Research by Gu et al. involved the innovative application of an NIR-III laser. Photothermal penetration experiments verified its highly efficient photothermal capabilities comparable to conventional NIR-I and NIR-II lasers. Compared to control groups and lower-dose NIR-III treatment (0.5 W cm^−2^, 5 min), NIR-III application (1 W cm^−2^, 5 min) resulted in complete tumor eradication (Gu et al., 2022).

#### 3.1.3 PTT and spinal metastases from *in situ* tumors, and brain metastases

Tumor metastasis presents a significant therapeutic challenge. Spinal metastases are a frequent complication in advanced lung cancer, while brain metastases affect many patients with various primary cancers. These secondary growths cause severe clinical issues and worsen patient prognoses (Hayat et al., 2007; Li et al., 2014; Zakaria et al., 2020; Rahman et al., 2023).

To address this, researchers are developing targeted nanoplatforms. One system, AMMD, uses a peptide to achieve high affinity for tumor cells, improving drug accumulation. It enables a combined photothermal and drug co-delivery strategy for spinal tumors. The specific drugs include phosphoinositide 3-kinase (PI3K) inhibitors and chemotherapeutic agents. The photothermal effect also enhances the drugs’ anti-osteolytic efficacy, reducing microstructural damage to the invaded spine (Ma et al., 2021). In other strategies, PTT can stimulate copper ion release, initiating a Fenton reaction. This elevates ROS levels, oxidizes DNA and proteins, and repolarizes macrophages. Such events trigger the ICD effect, activating anti-tumor immunity and consequently inhibiting tumor development, brain metastasis, and postoperative tumor spread (Tong et al., 2023).

### 3.2 PTT for neurodegenerative diseases

#### 3.2.1 PTT and Alzheimer’s disease

AD is the most prevalent neurodegenerative condition in older adults, comprising approximately 70% of dementia diagnoses. Projections suggest the number of affected individuals could surpass 100 million by 2050 (Huang D. et al., 2023). Although its complete pathogenesis is not fully understood, two primary pathological events are recognized: the extracellular deposition of Aβ aggregates and the intracellular formation of neurofibrillary tangles (NFTs) (Hamley, 2012; Lv et al., 2019; Sudhakar and Mani, 2019; Liu W. et al., 2021; Zeng et al., 2021). Aβ oligomers trigger both ROS generation and inflammation. They also activate mitochondrial death pathways, leading to tau protein hyperphosphorylation. Hyperphosphorylated tau subsequently detaches from microtubules, aggregates into NFTs, and culminates in neuronal death (Lv et al., 2019).

A central therapeutic goal involves hindering Aβ accumulation and eliminating existing protofibrils (Ling et al., 2021; Zeng et al., 2021). Given that amyloid formation is strongly temperature-dependent, PTT presents a viable approach (Liu W. et al., 2021). Localized heat generated by PTT can disintegrate Aβ aggregates and disrupt the stable physiological conditions necessary for their formation (Zeng et al., 2021). For instance, Song et al. demonstrated that Prussian blue-based nanomaterials under NIR radiation could dissociate Aβ fibrils (Song X. et al., 2023). Ge et al. introduced a CoP co-catalyst whose photothermal properties aid in diminishing Aβ deposition. Concurrently, it improves the photocatalytic efficiency of hydrogen evolution, producing H_2_ to selectively scavenge detrimental ROS, such as ·OH (Ge et al., 2023).

In addition to amyloid clearance, PTT can enhance BBB permeability during treatment (Zhou et al., 2020; Ge et al., 2022; Qi et al., 2023; Ye et al., 2023; Chi et al., 2024; Liu et al., 2024a). Enhanced permeability creates favorable conditions for drugs and nanocarriers to cross the BBB and reach Aβ protein aggregation sites. A study combining PTT with chemotherapy effectively opened the BBB, increasing substance accumulation within the mouse brain (Qi et al., 2023). Moreover, the photothermal effect can function as a “switch” to control the release of therapeutic drugs (Ling et al., 2021). Research using NGF-PCM@Ru NPs showed that drug release at 42°C was 3.7 times greater than at 37°C (Zhou et al., 2020). These properties suggest a broad potential for PTT in future clinical practice of AD.

#### 3.2.2 PTT and Parkinson’s disease

PD is characterized by dopaminergic neuron death, which depletes striatal dopamine and promotes α-synuclein aggregation (Goedert, 2015). PTT presents a promising strategy by enhancing nanomedicine delivery across the blood-brain barrier and enabling selective drug release via NIR light. This approach boosts the efficacy of medications like minocycline in clearing α-synuclein clumps (Li T. et al., 2021; Cheng et al., 2023). A mild photothermal effect offers synergistic neuroprotection by scavenging surplus ROS, mitigating neuroinflammation, and diminishing pathogenic protein buildup. Such actions help restore striatal dopamine, improve neurotransmitter signaling, and relieve motor impairments in PD mouse models (Xiong et al., 2020; Cheng et al., 2023). NIR irradiation amplified the neuroprotective capacity of BP-MT by facilitating matrine release and accelerating ROS clearance (Cheng et al., 2023).

Notably, minocycline, as part of a synergistic chemical and photothermal treatment platform, shows potential for treating other neurodegenerative conditions like amyotrophic lateral sclerosis and AD. GDY Nanosheets can be loaded with agents like SOD1 or chemical inhibitors targeting Aβ aggregation, allowing for NIR-controlled systemic administration and release (Li T. et al., 2021).

#### 3.2.3 PTT and Huntington’s disease

Huntington’s disease is a severe genetic disorder. It causes psychological instability, along with motor and cognitive decline (Ross and Tabrizi, 2011). The accumulation of large, insoluble protein aggregates is cytotoxic and represents a key therapeutic target. The buildup of protein aggregates can damage Huntington’s proteins essential for normal development (Truant et al., 2008; Arrasate and Finkbeiner, 2012). PTT can address these protein deposits. PTT disrupts existing aggregates through the photothermal treatment of unlabeled polyglutamine (polyQ), the coding trigger for the Huntington’s gene. It can also slow their formation. Furthermore, PTT could eliminate the protection against mutant Huntington’s proteins while producing thermal damage (Nedosekin et al., 2021). Additionally, PTT utilizes optical phenomena associated with the photothermal ablation process. The phenomena enable the visualization of protein aggregates. They also allow monitoring of the ablation process and control over treatment parameters (Nedosekin et al., 2021). High-resolution optical images documented aggregates in living worms before and after laser application. Post-treatment images revealed that some aggregates vanished, while others displayed significant alterations in shape, signifying thermal damage (Nedosekin et al., 2021).

## 4 Mechanisms of PTT application in neural tumors and neurodegenerative diseases

### 4.1 PTT initiates heat shock response

When NIR starts PTT of cancer cell regions with the help of PTA, the light-induced heat disrupts the integrity of the cell membrane, causing a large amount of Ca^2+^ to flow from outside the cell membrane into the cell, triggering chemical damage (Xie et al., 2020). When the tissue temperature exceeds 39°C, some proteins begin to aggregate and denature, and when the temperature rises to 41°C, a heat shock response is initiated, which induces rapid changes in gene expression patterns, of which heat shock proteins (Hsps) are important regulators to mitigate the effects of thermal injury (Jolly and Morimoto, 2000; Didelot et al., 2006; Li et al., 2020; Xie et al., 2020; Ren et al., 2022). Most Hsps are highly cytoprotective and can act as molecular chaperones for other cellular proteins. Upon severe exposure to high temperatures, heat-damaged proteins are sequestered through interactions with molecular chaperones and refolded to their natural state or degraded by the proteasome (Didelot et al., 2006). When cells are exposed to sublethal heat shock, they will temporarily acquire resistance to multiple stress conditions as well as multidrug resistance (Jolly and Morimoto, 2000). There is literature specifically designing photothermal therapies targeting Hsp70 to promote apoptosis in cancer cells (Ali et al., 2016), while SDT shows significant promise in synergistic PTT therapeutic applications due to its ability to downregulate the expression of Hsp90(Cao et al., 2022).

### 4.2 PTT induces apoptosis

PTT initiates apoptosis through multiple interconnected cellular stress responses. The thermal effects can compromise lysosomal membranes, leading to lysosomal membrane permeability. This allows tissue proteases to escape and activate pro-apoptotic proteins like Bid, which in turn exacerbates mitochondrial damage (Boya and Kroemer, 2008; Cao et al., 2021; Kadkhoda et al., 2022). Research confirms that thermal effects diminish the anti-apoptotic protein Bcl-2 while increasing the pro-apoptotic protein BAX (Liu et al., 2022). As mitochondria are particularly sensitive to heat (Kadkhoda et al., 2022), thermal stress also triggers mitochondrial membrane depolarization. This process results in the generation of ROS (Mocan et al., 2014), which inflict oxidative damage on proteins, lipids, and nucleic acids (Gorrini et al., 2013; Jaque et al., 2014; Ren et al., 2022). Studies on glioma indicate that this oxidative stress damages mitochondria, causing a loss of membrane potential and superoxide production that culminates in caspase-mediated apoptosis (Markovic et al., 2011). Similarly, the high temperatures from PTT can induce ERS. This response impairs the proper folding of proteins, and the resulting accumulation of misfolded proteins can ultimately trigger apoptosis in tumor cells (Gu et al., 2022).

The DNA damage caused by heat or ROS production activates the tumor suppressor p53, a key pro-apoptotic factor (Trachootham et al., 2009; Liou and Storz, 2010; Gorrini et al., 2013). Activated p53 can induce mitochondrial outer-membrane permeabilization (MOMP), which leads to apoptosome formation and initiates the apoptotic cascade (Evan and Vousden, 2001; Chipuk and Green, 2006; Trachootham et al., 2009; Ali et al., 2017; Gousias et al., 2022; Zhao et al., 2022). Experimental results support this, showing elevated p53 expression alongside reduced levels of Bcl-2 following PTT (Yao et al., 2022). Furthermore, p53 activation can deplete cell cycle regulators like p21, promoting G1/M or G2/M arrest and thereby inducing apoptosis (Hildebrandt et al., 2002; Gousias et al., 2022; Yu et al., 2022). Beyond these general pathways, PTT can be directed at specific oncogenic signaling loops. In glioblastoma, for example, the overexpressed oncogene MSH6 forms a feedback circuit with CXCR4 and TGFB1. This loop accelerates gliomagenesis, proliferation, and invasion while providing anti-apoptotic effects. Researchers have identified this specific oncogenic circuit as a potential therapeutic target for PTT in treating this cancer (Chen et al., 2019).

**FIGURE 1:**
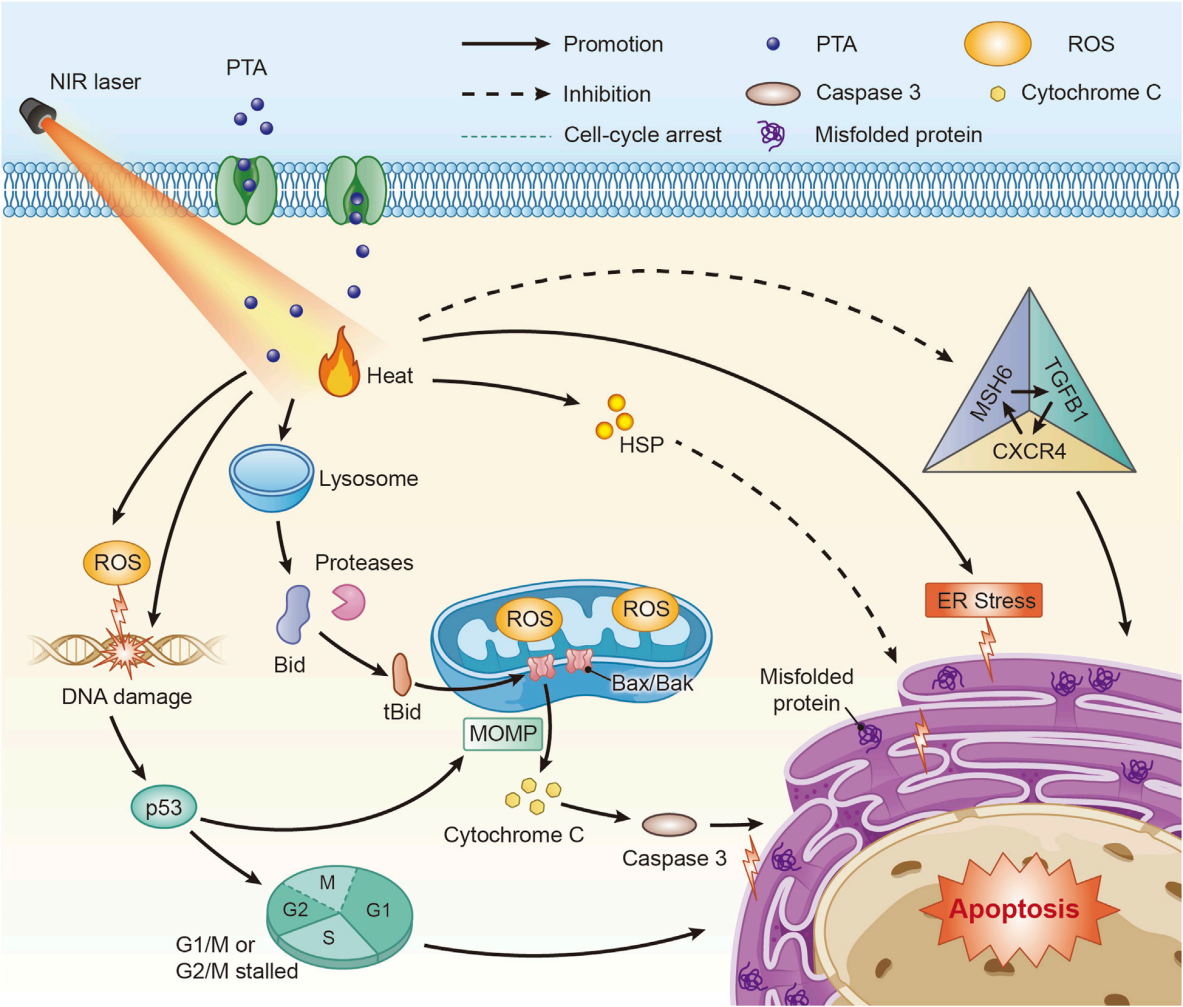
Molecular mechanisms of PTT associated with apoptosis in the treatment of neural tumors and neurodegenerative diseases. PTT can induce apoptosis through various molecular pathways such as p53 and ROS, among which the MSH6-CXCR4-TGFB1 feedback loop is a target used for PTT on GBM(Abbreviations: PTA: photothermal transduction agent, MOMP: mitochondrial outer-membrane permeabilization, ROS: Reactive oxygen species, HSP: heat shock proteins, ER: endoplasmic reticulum.)

The molecular pathways of apoptosis associated with PTT of neural tumors and neurodegenerative diseases are illustrated in Figure 1.

### 4.3 PTT enhances BBB permeability

The BBB is a physiological feature within the cerebral vasculature. It effectively regulates the passage of ions, cells, and molecules between the blood and brain tissue. The BBB also maintains the stable microenvironment crucial for neuronal signaling (Thangudu et al., 2020). However, protective function poses a significant challenge for designing therapies. The barrier’s capacity to prevent foreign substances from entering the brain impedes treatment delivery for brain disorders. Encouragingly, considerable research indicates PTT can enhance BBB permeability via the photothermal effect. This approach shows promise for treating AD (Zhou et al., 2020; Ge et al., 2022; Qi et al., 2023; Ye et al., 2023), PD (Cheng et al., 2023), depression (Zhang et al., 2021), and other neural tumors and neurodegenerative diseases.

### 4.4 PTT utilizes the photothermal effect to selectively release drugs and promote drug absorption

PTT serves as an external stimulus. It facilitates drug release specifically at the lesion site with temporal and spatial control. Photothermal effects can initiate drug release through various mechanisms, including the disruption of the drug carrier or heat-induced structural changes in sensitive materials. Alternatively, heat-responsive chemical bonds may break, or drug-carrier interactions can be destabilized. Furthermore, adjusting the laser intensity permits regulation of the drug release rate (Rai et al., 2010; Cheng et al., 2014; Liu et al., 2019b; Xie et al., 2020; Zhang et al., 2022a). Mild localized heat (≤43°C), generated by the nanomaterial template, also boosts the fluidity of the cell membrane. This increased fluidity helps nanomaterials penetrate cell or endosomal membranes more readily. Consequently, moderately high temperatures (40°C–43°C) during PTT can enhance cellular uptake. The enhancement results from increased cell membrane permeability (Cheng et al., 2014; Kim et al., 2016).

### 4.5 PTT improves intra-tumoral blood supply

PTT heating elevates tumor blood flow. It also increases endothelial gaps within the tumor vasculature and improves hemoglobin oxygen saturation (Jaque et al., 2014; Liu et al., 2019b; Overchuk et al., 2023). Elevated blood flow contributes to greater microvascular permeability, which broadens the therapeutic options for cancers resistant to penetration. Enhanced permeability facilitates better drug delivery and diffusion (Cao Y. et al., 2023). As a result, the effectiveness of chemotherapeutic agents against cancer cells is amplified (Dong et al., 2016; Liu et al., 2019b; Xu and Liang, 2020). Increased oxygen saturation within blood vessels can lessen tumor hypoxia. The improvement of the hypoxic microenvironment may counteract tumor drug resistance (Liu et al., 2019b; Xu and Liang, 2020). Improved tumor oxygenation can render the tumor more susceptible to radiotherapy and chemotherapy (Hildebrandt et al., 2002; Jaque et al., 2014; Liu et al., 2019b; Xie et al., 2020).

### 4.6 PTT utilizes the photothermal effect to promote gene transfection

The photothermal effect can disrupt endosomal membranes, which could promote endosomal escape, aiding cytoplasmic gene delivery in PTT/gene therapy strategies. Normally, cells internalize nucleic acids carried by nanoscale vectors via cytosis. The uptake mechanism produces endosomes that typically fuse with lysosomes. Photothermal facilitation helps therapeutic agents escape from endosomes before the fusion occurs (Kim et al., 2016). Additionally, short double-stranded DNA or RNA can be attached to metal nanoparticles. If the heat generated by PTT is adequate to melt these nucleic acids, it induces their de-hybridization. Consequently, the nucleic acids are released from the nanoparticle surface. The process achieves photothermal-induced gene release and enhances transfection efficiency (Kim and Kim, 2014; Hu et al., 2016; Kim et al., 2016; Xie et al., 2020).

### 4.7 PTT improves the efficiency of Fenton and Fenton-like reactions

CDT utilizes transition metal ions, including Fe^2+^, Co^2+^, Cu^+^, and Mn^2+^. These ions facilitate Fenton or Fenton-like reactions. Such processes convert H_2_O_2_, often overexpressed in the lesion’s cellular microenvironment, into ROS. The resulting ROS exhibit high toxicity towards cells (Qiao et al., 2022; Zhang et al., 2022a). Additionally, the photothermal effect associated with PTT can boost the efficiency of Fenton and Fenton-like reactions. The enhancement consequently improves the overall therapeutic efficacy of CDT (Pandey et al., 2020; Guo et al., 2022; Zhang et al., 2022a; Cao Y. et al., 2023).

### 4.8 PTT induces an immune response in the body

PTT ablates cancer cells, triggering ICD. Necrotic tumor cells then discharge various molecules, including damage-associated molecular patterns, TAAs, neoantigens, and pro-inflammatory cytokines. These released substances activate specific anti-tumor immune responses. Such responses involve the activation of immune effector cells and the promotion of T-cell infiltration. Cytokine secretion is also increased. Additionally, acute inflammation results from secreted pro-inflammatory factors and the infiltration of tumor immune cells. The inflammatory state fosters the activation and recruitment of dendritic cells (Xu and Liang, 2020; Zhao et al., 2022; Overchuk et al., 2023). In a similar way, PTT can modulate antiviral immune responses when confronting viruses. The process is achieved through the massive production of inflammatory factors, such as IL-6 and TNF-α during virus inactivation (Bai Y. et al., 2023).

**FIGURE 2:**
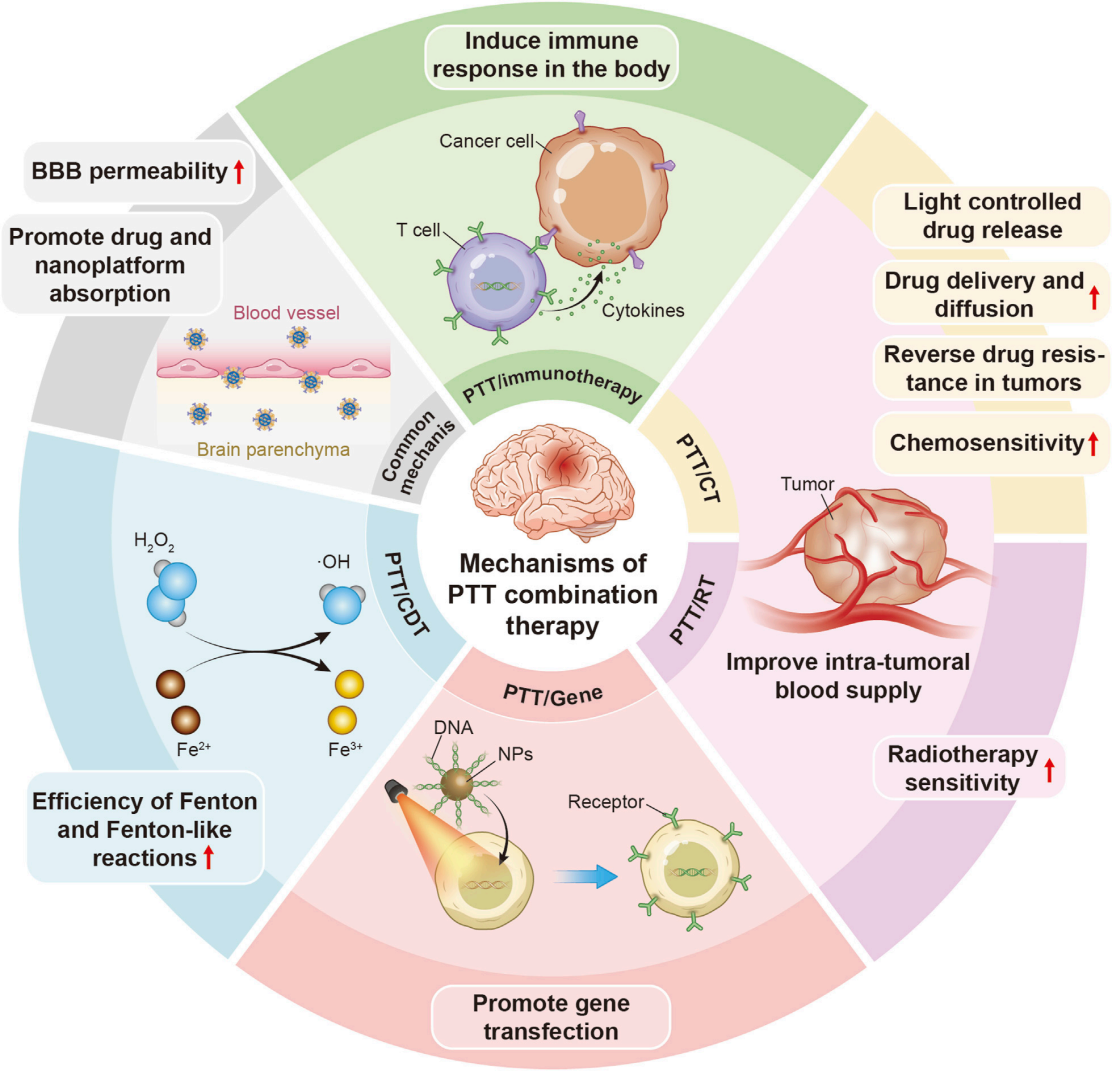
A schematic illustration of common mechanisms of PTT in combination with other therapies in the treatment of neural tumors and neurodegenerative diseases.

The common mechanisms of PTT in combination with other therapies in the treatment of neural tumors and neurodegenerative disorders are illustrated in Figure 2.

## 5 Advantages and disadvantages of PTT in neural tumors and neurodegenerative diseases

PTT has demonstrated multidimensional advantages in the treatment of nervous system diseases, not only contributing to the innovation of therapeutic strategies, but also greatly enhancing the safety and efficacy of treatment. The synergistic application of PTT and chemotherapy, through targeted delivery of drugs, significantly reduces systemic toxicities and side effects (Wang et al., 2014; Ma et al., 2020; Newland et al., 2022), achieves the precision of treatment and reflects the synergistic principle of “1 + 1>2” (Wang et al., 2014; Lan et al., 2021; Lu et al., 2021; Song Z. et al., 2023), which is also applied in its multimodal combination with PDT (Keyvan Rad et al., 2018), radiotherapy (Kargar et al., 2018; Yin et al., 2022), gene therapy (Hu et al., 2016) and so on. The integration of PTT technology with advanced imaging technologies (e.g., PAI, MRI, FLI) provides real-time, high-precision visualization guidance for surgical operations, which greatly improves the precision and safety of brain tumor surgery (Jin et al., 2017; Hu et al., 2018; Qian et al., 2018; Wang S. et al., 2021; Li Y. et al., 2022; Zhang et al., 2022a).

Further, by stimulating the immune system and triggering innate and adaptive immune responses against tumor cells, PTT not only directly inhibits tumor growth, but also effectively prevents tumor recurrence and thus improves the prognosis of tumors, demonstrating its potential in regulating the body’s immune response (Liu et al., 2019c; Huang et al., 2021; Sun et al., 2022). In addition, PTT facilitates the delivery of therapeutic drugs and platforms for neurological diseases by improving the permeability of the BBB, which covers a wide range from neurological tumors (Cao Y. et al., 2023) to neurodegenerative diseases (e.g., Alzheimer’s disease (Zhou et al., 2020; Ge et al., 2022; Qi et al., 2023; Ye et al., 2023)). Particularly important is that PTT utilizes photothermal response properties to achieve intelligent controlled release of therapeutic drugs, further enhancing the relevance and efficiency of treatment (Agarwal et al., 2011; Wang et al., 2013; Duan et al., 2017; Zhang et al., 2019; Li T. et al., 2021; Ling et al., 2021; Zhang et al., 2021; Wu F. et al., 2022; Manivasagan et al., 2023; Tong et al., 2023).

Although PTT has demonstrated significant advantages in the treatment of nervous system diseases, it still faces multiple challenges that require in-depth investigation and strategy optimization. The first problem is the limitation of NIR light penetration into tissues, which limits the depth of the photothermal effect and the efficiency of drug delivery, thereby affecting the intensity and reliability of the treatment (Lu et al., 2014; Wang et al., 2014). In addition, the heat shock reaction triggered at the initial stage of PTT may enhance the stress adaptation and drug resistance of cancer cells, thus hindering the effectiveness of the treatment (Jolly and Morimoto, 2000). PTT in photothermal ablation causes cellular necrosis, and thus induces inflammation. Inflammation can increase neutrophil levels and form neutrophil extracellular traps, further awakening dormant cancer cells (Liu Y. et al., 2021). In contrast, MPTT induces apoptosis of intact cell membranes, which prevents the inflammation caused by cell necrosis (Liu Y. et al., 2021).

Excessive or continuous heating during PTT may lead to vascular collapse and diminished tumor perfusion (Overchuk et al., 2023). Consequently, treatment protocols require a careful balance of photothermal properties, individual patient differences, and other factors to guarantee both safety and efficacy. Furthermore, PTT can activate autophagy mechanisms. The finding suggests that incorporating anti-autophagy strategies might be essential for improving therapeutic results (Zeng et al., 2022; Li J. et al., 2023). The advancement and use of nanomaterials also necessitate continuous enhancements in biocompatibility, cytotoxicity, and BBB penetration to realize optimal therapeutic performance (Cheng et al., 2014). It is important to recognize that PTT-induced ERS encounters strong resistance in certain malignant peripheral nerve sheath tumor cells, such as STS26T cells. The resistance arises from these tumor cells’ unique endoplasmic reticulum compositions, which permit increased expression of endoplasmic reticulum molecular chaperones and boost the degradation of unfoldable proteins, thereby creating a potent protective effect (Gu et al., 2022). Additionally, the effectiveness of PTT can be episodic and short-lived, especially when addressing protein aggregates in neurodegenerative conditions. This indicates that therapeutic strategies might need to be combined with long-term management programs to overcome the limitations of short-term efficacy and secure lasting therapeutic benefits (Nedosekin et al., 2021).

Nanotherapeutics for the central nervous system pose considerable neurotoxicity and safety risks. Some inorganic materials are directly toxic; silver nanoparticles provoke cerebral oxidative stress and neuroinflammation. Iron oxide nanoparticles can gather in microglia, causing cytotoxicity by releasing iron and generating reactive oxygen species (Singh et al., 2025). Dendrimers may also trigger adverse immune reactions. A particle’s physical properties, like larger dimensions or a positive surface charge, often elicit stronger immune responses (Vashist et al., 2023).

Methods for breaching the BBB could introduce further dangers. Opening the barrier can cause neurobehavioral side effects and allow circulating toxins into the brain (Kaushik et al., 2018; Thangudu et al., 2020). Moreover, the continuous NIR exposure associated with PTT produces excessive heat, which may damage healthy neural tissue. This poses a critical concern in non-cancerous neurological conditions and underscores the need for precise temperature control (Sagar and Nair, 2018; Liu et al., 2020; Zhao et al., 2021; Bai X. et al., 2023; Zeng et al., 2023).

## 6 Conclusions and perspectives

The convergence of nanotechnology and PTT offers a new therapeutic paradigm for the treatment of neurological diseases characterized by high disability and low efficacy. A unique aspect of this review is the systematic integration of multiple PTT nanomaterial platforms currently applied in neural tumors and neurodegenerative diseases. It covers different types of carriers such as organic systems (e.g., liposomes and peptide-based nanomaterials) and inorganic materials (e.g., gold and copper nanomaterials). The review focuses on analyzing the design principles of these materials for nervous system applications and exploring how they can be engineered to cope with the pathophysiological characteristics and biobarrier issues that are peculiar to nervous system diseases. A key part of this analysis involves comparing the organic and inorganic platforms based on their respective biocompatibility and biodegradation profiles, the scalability of their production, their overall cost-effectiveness, the regulatory obstacles and prospects for clinical translation. By comparing material selection and design strategies in different disease models, we provide a broad and deep perspective, emphasizing the important role of material innovation in enhancing the efficacy of photothermal therapies and expanding their potential applications in neurological disease treatment.

Crucially, the review also presents a comprehensive overview of the multifaceted clinical mechanisms utilized by PTT in these neurological contexts, including direct cell killing pathways (e.g., apoptosis), microenvironmental modulation (e.g., BBB permeability, vascular effects, drug/gene delivery augmentation), chemotherapy augmentation (e.g., Fenton’s response), and stimulation of host immunity.

PTT has demonstrated broad potential for treating nervous system diseases. The technique is minimally invasive, highly specific, and offers spatiotemporal selectivity (Huang and Lovell, 2017). PTT is particularly useful for the photothermal ablation of tumor cells. It is suitable for preoperative preparation, adjuvant surgery, and managing postoperative residual lesions (Gu et al., 2022). Additionally, optimized NIR lasers could provide feedback on the therapeutic effect for tumors. Among these, the NIR-III laser demonstrated higher efficiency than NIR-I in treating malignant peripheral nerve sheath tumors (Gu et al., 2022). For AD, combining PTT with Aβ inhibitors is considered a potentially better treatment strategy. This is because PTT enhances inhibitor efficacy, and light-responsive drug release systems reduce inhibitor side effects (Liu W. et al., 2021). Similar reasoning could address strong resistance induced by ERS. Therefore, it is hypothesized that combining NIR-III laser therapy with ERS inhibitors, like epimedin or growth hormone-releasing peptide, might improve therapeutic efficacy for certain malignant peripheral nerve sheath tumors (Gu et al., 2022).

The precision of PTT is further enhanced by various targeting strategies, such as dYNH-targeted peptides (Ma et al., 2021), RGD-targeted peptides (Huang et al., 2019; Song W. et al., 2023), pH-driven active targeting (Tong et al., 2023), laser targeting (Nedosekin et al., 2021), folate receptor targeting (Keyvan Rad et al., 2018), cellular markers (Zhang et al., 2019), cancer cell membrane (CCM) coating (Huang X. et al., 2023), immunological antibody-targeted photodetection (DeRussy et al., 2014; Li X. et al., 2021; Ren et al., 2021; Manivasagan et al., 2023) and bio-optical detection (Zhang et al., 2022b), which aim to achieve precise interventions for neurological diseases. Researchers are actively working to enhance the therapeutic impact of PTT. Efforts focus on improving the photothermal efficiency of photosensitizers. Specific strategies include modifying materials with hyaluronic acid and PEG (Cao et al., 2022). Others have combined gold nanoclusters with indocyanine green (Zhuo et al., 2022). Optimizing the density of zinc doping is also being explored (Li et al., 2019). Hydrogels have been combined with chitosan in several studies (Ko et al., 2021; Gao et al., 2022; Wu F. et al., 2022; Dong et al., 2023). Even the use of melanin derived from beards has been investigated (Zhang et al., 2021). Further research is anticipated to advance PTAs performance. Identifying chemicals that boost photothermal conversion is also a key goal.

Integrating advanced imaging with PTT is fundamental for improving therapeutic precision. Detailed anatomical information from MRI and computed tomography enables accurate tumor localization (Chen et al., 2019; Caro et al., 2021), while PAI provides real-time tracking of nanoparticle accumulation (Guo et al., 2017).

Finer procedural control is achieved using sophisticated methods. For instance, two-photon photoluminescence microscopy guides laser irradiation at the micro-level (Caro et al., 2021). Additionally, FRET-based nanoprobes offer sensitive, *in-situ* temperature feedback (Hu et al., 2018). To maximize specificity, activatable agents triggered by tumor biomarkers like elevated H_2_O_2_ or acidity confine the photothermal effect, sparing healthy tissue (Chen et al., 2017; Lee et al., 2024).

Repeated laser photothermal therapy may improve the transient nature of PTT, but this approach requires further experimental validation (Nedosekin et al., 2021). Notably, PTT displays potential for easing postoperative complications, such as peripheral nerve adhesions (Zhan et al., 2023). It can also modulate neurons for scientific research purposes (Zhao et al., 2019; Wu X. et al., 2022; Zhuang et al., 2023). Further investigations could examine PTT’s therapeutic impact on postoperative inflammation and residual lesions. Other potential areas include fever, infection, and related complications in diverse neurological disorders. Compared to traditional magnetothermal stimulation, PTT-induced neurostimulation eliminates the need for permanent brain implants or fibers in animals. It is free from size constraints and has a short excursion time. This positions PTT as a new method for controlling animal neuronal activity in research (Wu X. et al., 2022). Moreover, photothermal modulation affects neurons and muscle cells. This indicates potential applicability to other excitable cell types or experimental systems. It also introduces a fresh approach for the clinical management of neurodegenerative diseases and movement disorders. Additionally, it provides a novel strategy for clinical motor rehabilitation training (Zhuang et al., 2023).

It has been suggested in the literature that local heat generated during PTT triggers a heat shock response, thereby stimulating the production of Hsps (Zeng et al., 2021). Acting as molecular chaperones, Hsps regulate protein folding and activity, refolding proteins that have been misfolded or aggregated (Jolly and Morimoto, 2000; Didelot et al., 2006). The biological actions of Hsps could fundamentally reduce abnormal Aβ aggregation. This reduction may consequently mitigate AD. Nevertheless, research examining PTT’s impact on Hsps in AD remains scarce, awaiting further experimental development and investigation (Zeng et al., 2021).

Artificial intelligence, particularly its machine learning (ML) subset, offers a powerful strategy for nanomaterial design. ML provides efficient, controlled fabrication methods, circumventing the slow, resource-intensive nature of traditional synthesis (Tao et al., 2021; Rao et al., 2024). The approach involves two primary functions: prediction and experiment planning. Predictive models forecast nanoparticle characteristics from given synthesis conditions, while experiment planning algorithms intelligently guide the research process by suggesting subsequent experiments to efficiently identify optimal conditions. In parallel, planning algorithms like heuristic genetic algorithms or Bayesian optimization strategically guide research toward optimal parameters (Tao et al., 2021). Automated robotic and microfluidic platforms amplify these computational tools through high-throughput data generation. Integrating ML with such platforms creates closed-loop systems, accelerating optimization and facilitating novel material discovery (Rao et al., 2024).

## Glossary

AD: Alzheimer’s disease
BBB: blood-brain barrier
BP: Black phosphorus
CCM: cancer cell membrane
CDT: Chemodynamic Therapy
CQDs: carbon quantum dots
CT: Chemotherapy
DOX: doxorubicin
ERK: extracellular regulated protein kinases
ERS: Endoplasmic reticulum stress
FAPI: fibroblast activating protein inhibitor
FLI: fluorescence imaging
FLs: fluorescent poly-levodopamine nanoparticles
FRET: Förster resonance energy transfer
GBM: glioblastoma
Hb: hemoglobin
HGNP: Hollow gold nanoparticles
HRP: Horseradish peroxidase
Hsps: heat shock proteins
ICD: immunogenic cell death
ICG: indocyanine green
LCST: lowest critical solvation temperature
LSPR: localized surface plasmon resonance
ML: machine learning
MOF: metal-organic framework
MOMP: mitochondrial outer-membrane permeabilization
MPTT: mild photothermal therapy
MRI: magnetic resonance imaging
MSNs: Mesoporous silica nanoparticles
NFTs: neurofibrillary tangles
NGF: nerve growth factor
NIR: Near-infrared
PAI: photoacoustic imaging
PBNPs: Prussian blue nanoparticles
PD: Parkinson’s disease
PDA: Polydopamine
PDT: photodynamic therapy
PEG: polyethylene glycol
PTA: photothermal transduction agents
PTT: photothermal therapy
ROS: reactive oxygen species
RT: radiotherapy
SDT: Sonodynamic Therapy
SOD: superoxide dismutase
SPIO: Supramagnetic iron oxide
TA: tannic acid
TAAs: tumor-associated antigens
TMZ: temozolomide
TPE: main-chain tetraphenylene.
